# Integrated use of laboratory services for multiple infectious diseases in the WHO European Region during the COVID-19 pandemic and beyond

**DOI:** 10.2807/1560-7917.ES.2022.27.29.2100930

**Published:** 2022-07-21

**Authors:** Daniel Simões, Soudeh Ehsani, Maja Stanojevic, Natalia Shubladze, Gulmira Kalmambetova, Roger Paredes, Daniela Maria Cirillo, Ana Avellon, Irina Felker, Florian P Maurer, Askar Yedilbayev, Francis Drobniewski, Lara Vojnov, Anne S Johansen, Nicole Seguy, Masoud Dara, on behalf of the European Laboratory Initiative on TB, HIV and viral hepatitis core group members

**Affiliations:** 1 Instituto de Saúde Pública – Universidade do Porto, Porto, Portugal; 2 Member of the European Laboratory Initiative on TB, HIV and Viral Hepatitis (ELI) core group. The additional members of the ELI core group are listed under Acknowledgements; 3 WHO Regional Office for Europe, Copenhagen, Denmark; 4 Institute of Microbiology and Immunology, Faculty of Medicine, University of Belgrade, Belgrade, Serbia; 5 National Reference Laboratory, National Center for Tuberculosis and Lung Diseases, Tbilisi, Georgia; 6 National TB Reference Laboratory, Bishkek, Kyrgyzstan; 7 Infectious Diseases Department & irsiCaixa AIDS Research Institute, Badalona, Catalonia, Spain; 8 IRCCS San Raffaele Scientific Institute, Milan Italy; 9 Hepatitis Unit, National Center of Microbiology, Instituto de Salud Carlos III, CIBERESP, Madrid, Spain; 10 Novosibirsk Tuberculosis Research Institute, Novosibirsk, Russia; 11 National and WHO Supranational Reference Laboratory for Mycobacteria, Research Center Borstel, Borstel, Germany; 12 University Medical Center Hamburg-Eppendorf, Hamburg, Germany; 13 Infectious Diseases, Faculty of Medicine, Imperial College, London, United Kingdom; 14 WHO Headquarters, Geneva, Switzerland

**Keywords:** HIV, viral hepatitis, tuberculosis, sexually transmitted infections, laboratory services, integration

## Abstract

Technical advances in diagnostic techniques have permitted the possibility of multi-disease-based approaches for diagnosis and treatment monitoring of several infectious diseases, including tuberculosis (TB), human immunodeficiency virus (HIV), viral hepatitis and sexually transmitted infections (STI). However, in many countries, diagnosis and monitoring, as well as disease response programs, still operate as vertical systems, potentially causing delay in diagnosis and burden to patients and preventing the optimal use of available resources. With countries facing both human and financial resource constraints, during the COVID-19 pandemic even more than before, it is important that available resources are used as efficiently as possible, potential synergies are leveraged to maximise benefit for patients, continued provision of essential health services is ensured. For the infectious diseases, TB, HIV, hepatitis C (HCV) and STI, sharing devices and integrated services starting with rapid, quality-assured, and complete diagnostic services is beneficial for the continued development of adequate, efficient and effective treatment strategies. Here we explore the current and future potential (as well as some concerns), importance, implications and necessary implementation steps for the use of platforms for multi-disease testing for TB, HIV, HCV, STI and potentially other infectious diseases, including emerging pathogens, using the example of the COVID-19 pandemic.

## Background

The coronavirus disease (COVID-19) pandemic has added great strain to the health systems response on a global level [1,2]. Although at country level responses to the pandemic have varied, there was a common denominator in that no health system was prepared to respond to this threat without significant adaptations. In the World Health Organization’s (WHO) European Region, apart from public health measures to reduce transmission and managing capacity of hospital care, restructuring national testing and treatment monitoring for human immunodeficiency virus (HIV), tuberculosis (TB), viral hepatitis, and sexually transmitted infections (STI) and maximising the use of available resources in a short timeframe was a necessity in ensuring a swift response to the COVID-19 pandemic. Measures taken included reallocation of staff and equipment from TB, HIV, and other viral diagnostic services to SARS-CoV-2 testing [3]. Countries showed varying degrees of success in this restructuring and reallocation, depending on national infrastructure, staff skills and global shortages of key equipment, kits reagents and consumables [4,5]. In Eastern Europe in particular, microbiology and virology laboratories were often already under-resourced and were frequently overwhelmed [6] especially in earlier stages of the pandemic.

The WHO’s global [7-10] and regional [11-13] goals towards elimination of HIV, TB, viral hepatitis and STI are ambitious, and insufficient progress has been achieved in the last decade [14]. To reach these goals, enhancing integration of the existing testing and treatment monitoring systems for these diseases presents itself as a prime opportunity at various levels. Already before the COVID-19 pandemic, integration and decentralisation had been identified as a way forward in improving system capacity, efficiency and current service delivery models while providing more people-centred services [15-18]. Available evidence in multiple contexts includes, for example, data linkage showing ample opportunity for integrated service delivery [19] and increased case finding among key populations [20]. Feasibility studies show increased equipment usage upon integration, without compromising the original testing operations, and enabling swifter treatment initiation and access to viral load results for HIV [16,21]. Integrated testing has also been instrumental even for countrywide multi-disease elimination programs [22], despite few examples being available at this scale.

Under the current circumstances [1], it is important to be aware of existing opportunities for integration, and of the requirements to do so safely and successfully.

## The impact of the ongoing COVID-19 pandemic on integrated diagnostic services

Reallocation and redeployment of human and material resources within the COVID-19 response allowed countries to leverage existing know-how, equipment, facilities and workforce. However, as this was often achieved as a reaction to the requirements of responding to COVID-19, with little planning time, unintended consequences—such as decreasing capacity to respond to infectious diseases other than COVID-19 [23,24]—have contributed to the potential risk of loss of lives because of other diseases [25], especially in the initial stages of the pandemic.

Furthermore, adaptations made to respond to COVID-19 may have led to compromises in the quality of results because of increased workload of staff where laboratories were repurposed; lack of staff in cases where staff from TB, HIV, hepatitis and/or STI laboratories were repurposed to work on SARS-CoV-2 testing; lack of necessary reagents and consumables; and/or lack of necessary laboratory maintenance services. This was especially the case in the eastern part of the WHO European Region, where national capacity to perform services such as maintenance and certification of biological safety cabinets has been limited because of travel restrictions and/or high demand [6].

In some severe cases, the safety of laboratory workers may have been at risk due to these points as well as due to the lack of adequate personal protection equipment (PPE), disinfecting materials and/or employment of laboratory workers with lack of adequate knowledge, experience, and training in necessary biosafety measures.

## Vertical responses to TB, HIV, viral hepatitis and STI

Despite undergoing reviews and restructuring over time, disease response systems for HIV, TB and viral hepatitis remain mostly in vertical programs [26], with communication between disease areas at the programme management level, as well as between management and technical response infrastructures, considered to be low.

Although both the WHO Regional Office for Europe and the European Centre for Disease Prevention and Control (ECDC) have moved towards integration [27-30], this had so far limited effect on programmatic organisation for HIV, TB, viral hepatitis and STI nationally, including increasing the still limited dialogue among and between disease programs, and across the different programmatic levels [28].

In many settings, response to HIV, TB, viral hepatitis and STI is partly dependent on donor funding that is often project-related and focused on a particular disease. Donor agencies should further incorporate integrated approaches in funded projects, contributing to support and scale-up integration of laboratory services in national settings. Prior to the pandemic, in many countries, national responses to HIV, TB, viral hepatitis and STI were already struggling with available budgets, a situation that will have worsened with the added requirements of responding to the COVID-19 pandemic, making it difficult to reinforce the budgets of national responses to any of the infections. The global economic crisis [31,32] faced by all countries will further contribute to these budgetary restrictions in the coming years. In several Eastern European countries where the Global Fund has transitioned or is transitioning out, and thus where national governments will have to take over HIV and TB service delivery with domestic funding in a short timeframe, there will be additional challenges in implementing a high quality COVID-19 response, while ensuring that funds are in place to guarantee essential services are not interrupted or discontinued for HIV and TB.

## Integrating stakeholders in planning and implementation

As changing systems is a complex task and integrating diagnostic responses for multiple diseases can have considerable implications on testing systems, it requires careful and well-informed planning and management to be done effectively to mitigate inherent risks that are associated with changing processes [33,34]. To maximise the chance of success, evidence suggests the importance of: (i) adequate planning and justification for the integration, (ii) extensive and continuous communication regarding the purpose and expected changes, (iii) participatory co-creation of the vision, (iv) a dedicated change management team and adequate funding, including training and capacity building for new roles and responsibilities and (v) continuous monitoring of the implementation process and periodic evaluation of its impact [34].

Planning transitions towards integrated services thus needs to involve not only programme managers, but also laboratory directors and technicians, clinicians, primary healthcare workers, transportation companies, community organisations, service users and key patient populations to benefit from the experience and knowledge of those who use and operate the system. Similarly, transparent communication with representatives of these stakeholders can help reduce resistance to implementation, as well as encourage collaboration on planning and assessment stages.

In parallel, estimates of expected testing volumes and required staff time to deal with new testing requirements must inform the transition process and be followed by staff reinforcements or redeployments when necessary underpinned by appropriate cost-effectiveness analyses, to ensure that integration does not delay standard testing operations, or compromises the quality of the testing response.

## Inter- and intra-sectorial collaboration – integrated management and cooperation

Improving communication between stakeholders in the diagnosis and disease monitoring networks for TB, HIV, viral hepatitis and STI, including among and between disease programs and service delivery structures is critical and may lead to the identification of collaborative opportunities or synergies. Sharing equipment, staff and overall operational costs among different disease programs can also help make up for limited capacity to invest in single disease interventions and enable testing responses for multiple infections to be implemented at lower costs. For example, cost sharing or joint procurement of equipment, reagents, consumables, staff, and service and maintenance across disease programs or even countries can leverage price reduction.

However, as vertical programs tend to also have vertical budgets, combining procurement or implementing resource- or cost-sharing solutions is generally difficult, and in some cases impossible. Thus, coordination of planning processes is a more obvious first step to pave the way for integration at an implementation or procurement level. Countries where procurement is laboratory-based (or bottom-up) operate more integrated microbiology departments (e.g. the United Kingdom, Ireland, France, and the United States).

Because of limitations in the availability of staff, equipment or reagents in countries where diagnostic and disease monitoring services for HIV, TB, viral hepatitis, and STI are anchored in specialised laboratories, samples for diagnosis or monitoring of each infection are often sent to different sites, with varying response times, and less efficient use of both human and financial resources in the process. These also translate into complicated patient pathways and extended timeframes for availability of results. Relevant staff will already have received at least basic training in quality assurance and biosafety of specimen referral, and the vehicles themselves are likely to be easily converted for multiple specimen transport, offering an opportunity to increase the efficiency of sample transportation systems.

Analysis of the current sample referral and transportation systems by representatives from different disease programs might enable transportation of multiple sample types within the existing network, as well as consolidate a more comprehensive network of structures to conduct multiple assays, while reducing total transportation times and cost [35]. This can be achieved with little or no adjustment of national sample transportation guidelines and practices.

## Integrating available options and maximising the use of multi-disease testing platforms

Making the best selection from existing options in terms of testing services at national and subnational level is a crucial step to improve real world access to testing services; this choice will be influenced by considering individual and wider technical preferences in terms of schedules, location, patient acceptability and access, and overall cost regarding HIV, TB, viral hepatitis and STI testing.

Because of the technical requirements and complexity of diagnosis and monitoring, only a limited number of specific assays are viable for use in different levels of the system [36,37]. This means that more complex processes as well as high-throughput equipment will remain in specialised structures, but also that a series of essential assays can be run in a wider variety of settings with lower technical and infrastructural requirements, allowing for the provision of integrated services at closer proximity to users. For example, multi-disease rapid diagnostic tests (RDTs), point of care (PoC) or near-PoC portable platforms can enable TB, HIV, viral hepatitis and STI diagnosis to be implemented at additional points in the testing system and closer to patients. These can be used as near-PoC systems allowing local hospitals, small clinics, harm reduction services, primary healthcare units and even community-based organisations to provide some diagnosis and treatment monitoring options for HIV, TB, viral hepatitis or STI closer to where people and affected communities are.

In parallel, allowing for all relevant tests to be performed in as many sites as possible will maximise contact with services, providing a health response adjusted to the local epidemiology and the needs of populations served, while simplifying patient pathways and possibly decreasing the number of visits and overall time for diagnosis of multiple infections.

## Integrating other services with testing and treatment monitoring

Taking into account the fact that many patients, particularly within the key populations, have coinfections and that laboratory services may not be easily accessible for them, bringing testing and disease monitoring closer to the people also facilitates access to complementary services. Here the role of local clinics, primary healthcare units, harm reduction centres, community organisations and health centres for detainees in linking people coming for testing or treatment monitoring to other essential services cannot be dismissed. Ideally, diagnostic integration would evolve to service and care integration for patients so they can access all necessary services across diseases through simplified and integrated service delivery.

Furthermore, ensuring access to support services adjusted to the needs of the users in areas such as housing, food security, employment, or social security, can provide more person-centred and holistic services. Existence of referral networks to other services, or services available on site may also increase such test and care seeking.

## System and workforce preparedness

Investing in training and having well trained staff including in multi-disease testing platforms has the advantage that knowing how to run the platform often enables the person to conduct multiple assays, provided specimens are available and the relevant training (e.g. on biosafety) is made available. Training in different specimen handling, as well as knowledge on necessary biosafety measures for the different infectious sample types, increases adaptability of staff and facilitates redeployment or reallocation of staff when necessary. Staff training in different techniques increases capacity and flexibility of laboratory response to workflow changes, and integration may thus translate into improved capacity to better manage shifts in testing flow at a site level and relocating staff from one site to another.

At a site level, working with new sample types, implementing new tests and equipment, and communicating these results will require the implementation of standard operating procedures (SOPs), adequate biosafety measures, development and delivery of staff training, and creation or adaptation of sample transportation networks, among others. Regardless of the setting, existing biosafety training can be quickly adapted if necessary or replicated for the staff that will be working with new sample types or tests, and the same should be applicable for SOPs.

Qualified staff within the system should be able to rapidly adapt training manuals and deliver training sessions in any topics that are not yet covered, and international guidance and collaboration can fill the gaps that may exist at a technical level.

## Discussion

Besides the obvious difficulty in changing processes in complex systems, the COVID-19 pandemic added difficulty to initiating and progressing with integration of HIV, TB, viral hepatitis and STI responses, and several key challenges need to be addressed.

Policymakers and health systems have been focused primarily in responding to the COVID-19 pandemic and minimising its impact, which decreases their interest to approach other disease areas and implement new interventions. This, coupled with the aforementioned reallocation of human resources at all levels of the systems as part of the COVID-19 response, has reduced the attention to other infectious diseases with less staff available to plan and support integration in those disease areas.

Political and institutional will and commitment are requirements to effectively implement structural changes. It is thus critical to leverage what seemed to be an environment of openness and solidarity, revealed by multiple reported changes – not always documented in research - in clinical service delivery models, particularly regarding HIV [38], and initiate or advance integration of disease responses, particularly those which require limited investment.

Some countries may find it difficult to capitalise on the opportunities provided by the current public health crisis due to their vertical programs having their own legal basis and earmarked funding source(s). Thus, it may not be feasible to integrate laboratory services across different vertical programs without integrating all aspects of these programs, which typically requires a ministerial decree and governmental approval, making integration of vertical programs both politically difficult and time-consuming. In addition, many low- or middle-income countries have inadequately developed health system stewardship functions (Box) and lack both the capacity to regularly monitor the performance of their health systems, as well as institutionalised processes and mechanisms to take corrective actions when assessments indicate that they are needed [39].

Box **Domains of health system stewardship**

- Maintaining the strategic direction of policy development and implementation
- Detecting and correcting undesirable trends and distortions
- Articulating the case for health in national development
- Regulating the behaviour of a wide range of actors from healthcare financiers to healthcare providers
- Establishing effective accountability mechanisms

Adapted from: World Health Organization. Health systems stewardship (https://www.who.int/healthsystems/stewardship/en).

The crisis caused by the COVID-19 pandemic has documented the importance of and the need to strengthen the institutional capacity and governance mechanisms of the ministries of health in many countries [40]. While this may be politically difficult and time-consuming, it should not be a barrier to carrying out needed reforms.

Conversely, the COVID-19 pandemic has offered a rare opportunity to initiate long-overdue reforms while also strengthening the stewardship capacity and governance mechanisms in the process; several lessons have been learned from this including for the laboratory setting, with the potential to improve response to other epidemics going forward [41-44]. If done well, this could have long-term benefits beyond improving the performance of the merged disease programs. Indeed, it could improve the performance of health systems more broadly, including improving disease surveillance at national and international level. To achieve this, it is crucial that systems are in place that ensure test results to be easily monitored and captured for surveillance purposes, making information management systems another crucial component—and often a neglected challenge—in integration processes.

The importance of carefully considering the workforce affected cannot be overestimated. Skilled workers are a scarce resource in all countries and if the integration process is not handled well, critical staff may leave, which can further affect and undermine the motivation of those who remain, and negatively impact their productivity. It is also important to consider that resistance because of fear of loss of jobs or benefits is possible in response to changes in any system, particularly when objectives and processes are not clear to all those that are involved or affected. Management of doubts, fears and uncertainties can be facilitated by having all relevant stakeholders involved in planning and implementing changes mapped to a clear vision of what is wanted at the end of the process of change.

Additionally, although opportunities may arise to increase efficiency at reduced cost, initially, integrating new tests, maximising use of equipment, and increasing available options for testing and treatment monitoring in any country will require adequate financial investment. Effective access to novel testing options (either equipment or assays) in all countries within the WHO European Region is an additional key step in ensuring equitable access to diagnostics.

## Conclusions

With timely diagnosis of HIV, TB, viral hepatitis and STI still lagging in the region, designing and implementing more accessible, swift and efficient diagnosis and disease monitoring responses is a crucial task in the years to come. With a wide range of structural barriers remaining worldwide and COVID-19 very likely not being the last unexpected emerging infectious disease, solutions towards integrated services that would allow agile diagnostic capacities is essential. The fact that the pandemic has left no other option in many countries and setting other than using their limited capacities—particularly with regards to laboratory services—in a more integrated manner can be seen as an opportunity towards the implementation of integrated systems. Successful integration requires that systems are well prepared, and transitions are done in a stepwise approach. In some countries, this might require deep changes to the current programmatic structure, which in turn would require changes to legal and funding frameworks. While likely to be politically challenging and both time- and resource-consuming, a long-term plan that takes managerial, financial, human resources and organisational aspects into account will lead to more sustainable changes towards integrated, person-centred services and systems, and may also contribute to improve global preparedness and the response to future pandemics.
